# Neuroprotective effects of electroacupuncture in ischemic stroke: from mechanisms to clinical implications

**DOI:** 10.3389/fnagi.2025.1562925

**Published:** 2025-04-24

**Authors:** Jinxi Zhu, Xinyi Ge, Yulong Cao, Renjie Xiao, Xiao Deng

**Affiliations:** ^1^Department of Operating Room, The Second Affiliated Hospital of Nanchang University, Nanchang, Jiangxi, China; ^2^Department of Anesthesiology, The Second Affiliated Hospital of Nanchang University, Nanchang, Jiangxi, China; ^3^Nanchang University Queen Mary School, Nanchang, Jiangxi, China

**Keywords:** ischemic stroke, cerebral ischemia/reperfusion injury, electroacupuncture, neural protection, autophagy, oxidative stress

## Abstract

Ischemic stroke is a condition caused by an interruption of blood flow to the brain that can lead to neurological damage. The severe neurological damage caused by an ischemic stroke can lead to cognitive impairment and even disability. Reperfusion therapy is the mainstay of treatment for ischemic stroke. However, while restoring oxygen and blood flow to the brain tissue can reduce or prevent neuronal cell damage and death caused by cerebral ischemia, ischemia/reperfusion may trigger pathological tissue reactions leading to neuronal cell damage. Excessive autophagy in neuronal cells, disruption of cellular oxidative homeostasis leading to oxidative stress, apoptosis, glutamatergic excitatory damage, ferroptosis, and neuroinflammation are all key pathways contributing to cerebral ischemia/reperfusion injury. Electroacupuncture, as an extension of traditional Chinese acupuncture, has obvious effects on alleviating cerebral ischemia/reperfusion injury. Many experiments have observed that after electroacupuncture treatment or pretreatment in rats, cognitive impairment was reduced, brain tissue morphology was improved, and the damage pathways such as autophagy, oxidative stress, neuroinflammation, and apoptosis were significantly inhibited, and the recovery pathways such as the blood-brain barrier and angiogenesis were significantly promoted. Although the specific mechanism of electroacupuncture therapy is not known, it has great potential in the treatment of ischemic stroke and cerebral ischemia/reperfusion injury. Electroacupuncture to improve cerebral ischemia/reperfusion injury is a new target for therapeutic approaches. In the future, electroacupuncture is expected to become an effective therapy for cerebral ischemia/reperfusion by conducting more clinical trials and enriching the understanding of its mechanism for improving cerebral ischemia/reperfusion injury.

## 1 Introduction

Stroke, a cerebrovascular accident, results from acute focal CNS damage and presents as neurological deficits (Hilkens et al., 2024). These deficits primarily stem from cerebrovascular anomalies that disrupt cerebral circulation. Global stroke prevalence has risen steadily, recording a 70% surge in incident cases between 1990 and 2019 (Bersano and Gatti, 2023; Feigin et al., 2022). China shows particularly concerning statistics, with age-standardized stroke incidence increasing by 11.5% during 1990-2021 (Krishnamurthi et al., 2020), highlighting ongoing challenges in national stroke management. Ischemic stroke constitutes 63% of total stroke cases (Potter et al., 2022). This condition features cerebral hypoperfusion, triggering tissue necrosis and vascular territory-specific neurological dysfunction. As a primary cause of cognitive impairment and disability (Zhao et al., 2022), ischemic stroke exerts significant socioeconomic burdens through high morbidity, recurrence rates, and mortality, directly compromising patient outcomes and straining public health systems (Tang et al., 2021).

Current therapies employ thrombolysis and thrombectomy to achieve cerebral reperfusion prior to irreversible neuronal damage (Shen et al., 2021). Paradoxically, this intervention triggers pathological cascades—including neuroinflammation (Candelario-Jalil et al., 2022), autophagy dysregulation (Du et al., 2020), and oxidative stress (Zhu et al., 2023), culminating in cerebral ischemia/reperfusion injury (CIRI). The pathophysiological complexity of CIRI involves interrelated mechanisms: reactive oxygen species (ROS) overproduction, calcium dyshomeostasis, glial activation, and apoptotic pathways (Wang Z. et al., 2023). These processes induce microvascular constriction, blood cell aggregation, cerebral edema, and neuronal apoptosis, synergistically exacerbating pathological cascades (Zhang et al., 2024), and impeding clinical management with compromised prognostic accuracy. Electroacupuncture (EA), an advanced acupuncture technique utilizing standardized electrical parameters, delivers consistent therapeutic stimulation with demonstrated safety (Franke et al., 2021). Preclinical studies validate EA’s neuroprotective effects against CIRI-induced neurological damage (Qi et al., 2022; Wang M. et al., 2020), though its mechanisms demand further elucidation. Current findings suggest EA modulates critical pathological processes including neuroinflammatory responses, oxidative balance, and apoptotic regulation. This review synthesizes evidence on these multifactorial mechanisms to optimize EA-based therapeutic strategies for CIRI.

## 2 Electroacupuncture

Acupuncture therapy functions via targeted stimulation of meridian pathways to trigger systemic physiological responses (Liu et al., 2021). EA augments this process by applying controlled electrical stimulation to acupoints, amplifying therapeutic effects. Preclinical studies validate its multifactorial efficacy: facilitating endogenous neural stem cell differentiation in post-stroke recovery (Zhang et al., 2021), modulating pain signaling in inflammatory conditions (Zhang Q. et al., 2023), and enhancing cognitive ability while improving neurological function in Alzheimer’s disease model animals (Zheng et al., 2021). These findings demonstrate EA’s unique capacity to target neuro-muscular pathologies. Clinical trials corroborate these preclinical findings, confirming EA’s translatable efficacy across neurological disorders (Mao et al., 2021; Table 1).

**TABLE 1:** Literature review on the effectiveness of EA.

Author	Year	Research objective	Acupuncture points	Frequency	Duration of intervention	Experimental/ control group	Assessment	More effective than the control	*P*
Kong et al. (2020)	2020	EA in chronic low back pain	KI3, KI7 HR3, SI3, BL40, GV3, GV20, KI10, BL10, BL40	2 Hz	6 weeks	EA/pseudo-EA	1. PROMIS score 2. RMDQ	+	1. = 0.02 2. = 0.02
Kang et al. (2023)	2023	EA in relieving acute pain after TKA	ST32, ST36, SP9, GB34	2 Hz	5 days	EA/SA	1. ALFF 2. NRS 3. SDS	+	1. < 0.05 2. < 0.001 3. < 0.05
Shaosong et al. (2024)	2023	EA in skeletal muscle pain in Parkinson0, Bisease	GV20, CV6, LU7, SI19	2 Hz/100 Hz alternately	4 weeks	EA/pseudo-EA	1. VAS 2. KPPS	+	1. < 0.05 2. < 0.05
Penza et al. (2011)	2011	EA in chronic painful neuropathies	ST36, SP6, LR3, BL60	2T36, S	24 weeks	EA/pseudo-EA	VAS	−	−
Yin et al. (2022)	2022	EA in comorbid insomnia and depression	GV20, GV24, GV29, EX-HN22, HT7, PC6, SP6	30 Hz	8 weeks	EA/SA	1. PSQI 2. HDRS 3. ISI	+	1. < 0.001 2. < 0.001 3. < 0.001

RMDQ, Roland Morris Disability Questionnaire; SA, sham-acupuncture; VAS, visual analog scale; TKA, total knee arthroplasty; ALFF, the amplitude of low-frequency fluctuation; NRS, numerical rating scale; SDS, Self-Rating Depression Scale; KPPS, Kingplitude of lowL40rough f ischemic stroke: an overview of cIndex; HDRS, Hamilton depression rating scale; ISI, Insomnia Severity Index.

Contemporary ischemic stroke management integrates pharmacological thrombolysis with mechanical interventions. First-line protocols prioritize intravenous tissue plasminogen activator (Zhao et al., 2022) supplemented by dual antiplatelet therapy (aspirin/clopidogrel) for minor strokes, while mechanical thrombectomy is reserved as the gold-standard intervention for disabling cases (Mendelson and Prabhakaran, 2021). EA emerges as a complementary modality, counteracting thrombolysis-associated ischemia/reperfusion injury through neuroprotective mechanisms. Its demonstrated safety profile, synergistic enhancement of conventional therapy efficacy, and minimal adverse effects establish EA as a strategic adjunct therapy for comprehensive cerebral injury management.

Despite demonstrated preclinical efficacy, EA’s clinical translation requires systematic validation through rigorously designed trials. Key research priorities center on elucidating disease-specific mechanisms across neurological conditions, optimizing stimulation protocols through parameter standardization, and establishing multi-dimensional safety assessments encompassing long-term outcomes. Addressing these challenges will enable the precise integration of EA into evidence-driven stroke rehabilitation frameworks, bridging the gap between experimental findings and clinical implementation.

## 3 The improvement of cerebral ischemia/reperfusion injury through electroacupuncture mechanisms

**FIGURE 1:**
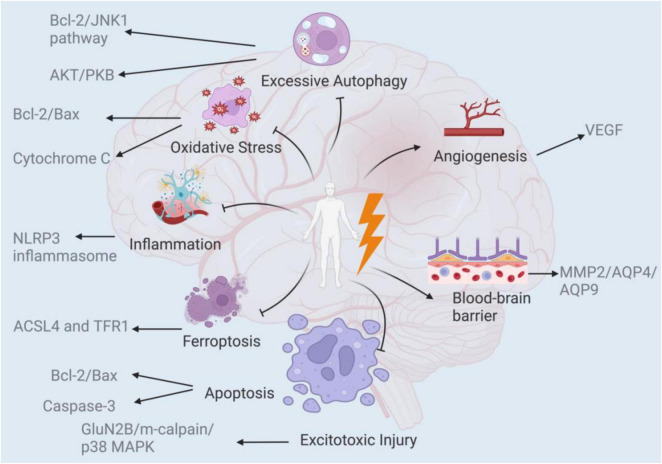
Several aspects of EA treatment/pre-treatment to improve CIRI.

EA ameliorates CIRI through dual mechanisms: injury reversal and neuroprotection. Post-injury, neuronal and glial cells exhibit pathological manifestations including inflammation, oxidative stress, excessive autophagy, ferroptosis, and excitotoxic injury. These processes collectively induce apoptosis and impair neurological function (Zhang et al., 2024). This review systematically elucidates the mechanisms underlying EA’s therapeutic effects on CIRI, specifically analyzing its dual regulatory pathways: pathogenic process suppression and neural repair activation (Figure 1).

EA works in a variety of ways to improve cerebral ischemia/reperfusion. It promotes the blood-brain barrier and angiogenesis against injury and prevents excessive autophagy, apoptosis, oxidative stress, inflammation, ferroptosis, and excitotoxic injury.

### 3.1 Inhibition effect

#### 3.1.1 Autophagy

Autophagy, an evolutionarily conserved eukaryotic cellular process, sustains intracellular homeostasis through macromolecule and organelle degradation. This mechanism employs autophagosomes and lysosomes to achieve dual biological outcomes: recycling of degradation products or induction of non-apoptotic programed cell death. Under pathological conditions, excessive autophagy may trigger cell death (Levine and Kroemer, 2019; Liu S. et al., 2023; Vargas et al., 2023; Zhang et al., 2022). Autophagy is functionally categorized into two subtypes: non-selective (bulk) autophagy and selective autophagy. The latter specifically mediates targeted degradation of individual cellular components, exemplified by mitophagy in mitochondrial clearance (Klionsky et al., 2021).

Current studies report conflicting findings regarding the neuroprotective effects of EA-mediated autophagy regulation during cerebral ischemia-reperfusion. In a recent investigation, Qiu et al. quantified autophagy markers (LC3 and p62) in CIRI rat neurons, observing that EA decreased LC3 expression and increased p62 expression. Researchers administered EA at Baihui, Quchi, and Zusanli acupoints with parameters: 2/15 Hz dense-dispersive wave, 1 mA current. Results indicate that EA suppresses autophagy during reperfusion, alleviating CIRI (Mizushima and Levine, 2020; Qiu et al., 2025).

Conversely, emerging evidence suggests autophagy upregulation exerts neuroprotective effects. Xu et al. demonstrated that EA treatment enhances autophagic activity and mitigates CIRI. EA was administered at Du20 and Du26 acupoints at 5 min and 16 h post-injury, employing sparse (3.85 Hz for the 1.28-s duration) and dense (6.25 Hz for the 2.08-s duration) wave patterns. Stimulation parameters comprised a current intensity of 0.8–1.0 mA delivered over a 30-min treatment session (Xu et al., 2020). The precise mechanisms underlying EA-mediated autophagy regulation during injury repair require systematic investigation. These divergent outcomes may stem from intercellular variations in baseline autophagy levels across experimental models. Specifically, in cellular populations with diminished autophagic activity, EA potentially enhances autophagic flux, facilitating the clearance of compromised organelles and subsequent mitigation of injury. Conversely, in cellular systems exhibiting elevated basal autophagic activity, EA may exert inhibitory regulation on autophagic flux, thereby attenuating pathological consequences associated with excessive self-digestive processes.

Cellular autophagy is modulated through multiple signaling pathways, with extensive research elucidating key regulatory mechanisms. However, the precise pathway by which EA exerts its autophagic regulation remains undetermined, attributable to limited mechanistic evidence. Current investigations primarily focus on two pathways: mTOR-dependent and mTOR-independent signaling cascades. The mTOR-independent cascade, canonically designated as the Beclin1/B-cell lymphoma 2 (Bcl-2)/c-Jun N-terminal kinase 1 (JNK1) signaling axis, operates through coordinated regulation by autophagy-related genes (ATG) (Ahsan et al., 2021).

EA ameliorates CIRI through mTOR pathway upregulation, modulating autophagy activity (Wang M. et al., 2020). mTOR functions through two distinct complexes: mTOR complex 1 (mTORC1) and mTORC2. mTORC1 primarily suppresses autophagic activity, whereas this regulatory effect operates through the class I phosphatidylinositol 3-kinase (PI3K-I)/protein kinase B (AKT) signaling axis. Class 1 PI3K initiates this signaling cascade by phosphorylating plasma membrane phospholipids, thereby activating AKT. Activated AKT subsequently phosphorylates tuberous sclerosis complex 2 (TSC2), disrupting the TSC1-TSC2 interaction and ultimately relieving mTORC1 from inhibitory regulation (Ahsan et al., 2021; Tian et al., 2022).

**FIGURE 2:**
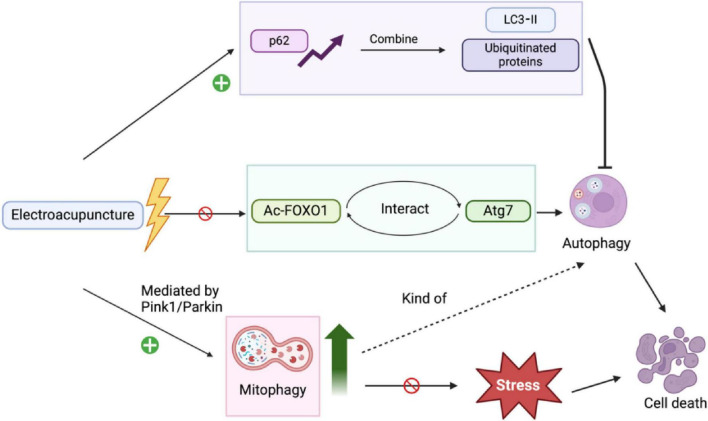
The pathways adjust autophagy/mitophagy to prevent cerebral ischemia/reperfusion injury. LC3-II, light chain 3-II; AC-FOXO1, acetylated FOXO1; Atg7, autophagy-related gene 7.

EA modulates autophagy through dual regulatory mechanisms: controlling autophagosome generation and upstream ATGs. Experimental evidence indicates that EA pretreatment suppresses LC3 accumulation while elevating p62 expression, concurrent with significant structural restoration of neurons in the ischemic cerebral cortex of rat models. The regulation of autophagosomal components constitutes a critical control node in autophagy modulation (Mei et al., 2020; Figure 2). Experimental evidence reveals that silent information regulator 1 modulates autophagic activity via deacetylation mechanisms targeting both histones and non-histone proteins, including FOXO1 and p53. Under cerebral ischemia-reperfusion-induced oxidative stress, cytoplasmic acetylated FOXO1 accumulates and interacts with ATG7, a core autophagy executor, thereby activating autophagic flux. EA intervention effectively reverses this molecular interaction cascade (Mei et al., 2020; Figure 2).

Mitophagy serves as a critical neuroprotective mechanism in EA-mediated mitigation of CIRI. Ischemia-hypoxia during injury, disrupts mitochondrial electron transport chain function, impairing oxygen/glucose supply and adenosine triphosphate (ATP) synthesis. This oxygen delivery-consumption mismatch compromises oxidative phosphorylation, amplifying neuronal degeneration. Prolonged hypoxia depletes ATP, disrupting energy-dependent ion channel function and triggering sequential pathological events: potassium efflux, membrane depolarization, and voltage-gated calcium channel activation. Subsequent intracellular Ca^2+^ overload induces mitochondrial edema, which initiates pro-apoptotic factor release and structural disintegration of mitochondria. During reperfusion, electrons escaping from the impaired respiratory chain react with molecular oxygen, generating ROS that potentiate mitochondrial damage through oxidative stress cascades. ROS mediate dual activation of AMPK and NF-κB signaling pathways, driving proinflammatory cytokine secretion while amplifying oxidative damage (Li and Xing, 2023). Autophagy can also aggravate oxidative stress injury, and the combination of these reactions worsens cellular damage. Experimental data reveal that 20 Hz compressional wave EA at Baihui (GV20) and Zusanli (ST36) acupoints for 30 min activates PTEN-induced putative kinase 1 (PINK1)/Parkin-dependent mitophagy, effectively counteracting nitro-oxidative stress-mediated mitochondrial dysfunction in CIRI (Wang et al., 2019; Figure 2). Pink1, a central regulator of mitophagy, undergoes activation in response to mitochondrial dysfunction, subsequently recruiting Parkin to orchestrate mitochondrial clearance through selective autophagy (Ji et al., 2022). The pathophysiological triad of oxidative stress, inflammatory cascades, and autophagic dysregulation exhibits reciprocal potentiation, forming a self-amplifying circuit that escalates cytopathological damage. Strategic therapeutic intervention targeting this interconnected network demonstrates enhanced potential for achieving multidimensional neuroprotection in ischemia-reperfusion pathologies. Dysfunctional mitochondria constitute primary generators of ROS in oxidative stress. Controlled mitophagy exerts cytoprotective effects by selectively eliminating compromised mitochondria and inhibiting ROS overproduction, effectively attenuating pathological oxidative stress propagation and downstream inflammatory cascades.

The precise mechanisms underlying EA dual modulation of autophagic activity and mitigation of autophagy-mediated cytopathology remain systematically uncharacterized. Current evidence suggests these regulatory processes exhibit inherent complexity and multifactorial interactions. Deciphering such mechanisms holds significant potential to enhance the efficacy of EA-based therapeutic strategies for CIRI. Targeted investigations are required to map the molecular circuitry governing autophagic regulation and its pathophysiological crosstalk in neural injury contexts.

Regulation of autophagy by EA is one of the main targets to improve CIRI, mainly by regulating the expression of autophagy-related proteins, and autophagy-related genes to achieve the prevention of damage produced by excessive autophagy in neuronal cells, but part of autophagy, such as mitophagy, is up-regulated by EA, and it can prevent the occurrence of oxidative stress.

#### 3.1.2 Oxidative stress

Oxidative stress manifests as a pathophysiological imbalance wherein pro-oxidant forces override antioxidant defenses, disrupting cellular redox equilibrium. In biological systems, endogenous antioxidant enzymes constitute the primary molecular machinery responsible for counteracting oxidative damage and maintaining metabolic homeostasis. Antioxidant defense mechanisms encompass multiple molecular regulators and signaling pathways, notably the nuclear factor E2-related factor 2, Kelch-like ECH-associated protein 1, PI3K/AKT cascade, mitogen-activated protein kinase, NF-κB inflammatory pathway, and heme oxygenase-1 enzymatic system, which collectively maintain redox homeostasis through coordinated transcriptional regulation and stress response modulation (Forman and Zhang, 2021; Wang et al., 2022b).

Oxidative stress plays a central role in CIRI; its pathological dominance constitutes a primary therapeutic target (Huang G. et al., 2022). CIRI induces redox imbalance through two principal mechanisms: ROS overproduction and compromised antioxidant enzymatic defenses. Pathological suppression of the Wnt/β-catenin pathway exemplifies this process, as it triggers mitochondrial complex I dysfunction and ROS overgeneration. This propagates mitochondrial destruction, culminating in programed neuronal death (Zhang et al., 2024).

**FIGURE 3:**
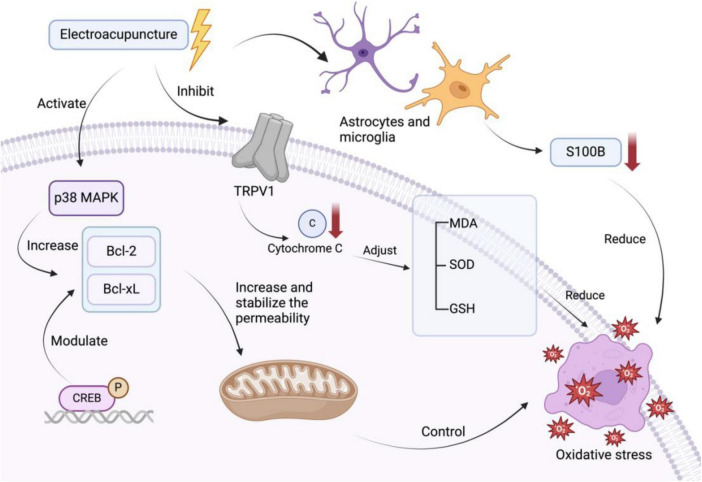
Several targets of EA to treat oxidative stress in cerebral ischemia/reperfusion injury.

EA demonstrates significant efficacy in ameliorating oxidative stress injury following cerebral ischemia-reperfusion (Tang et al., 2024). Experimental studies reveal that EA preconditioning attenuates redox imbalance through coordinated suppression of transient receptor potential vanilloid 1 activation, downregulation of cytochrome C, and biomarker regulation of MDA, glutathione (GSH), and SOD levels (Figure 3; Long et al., 2019). Cheng et al. demonstrated that MAPKs serve as central regulators of intracellular oxidative stress post-cerebral ischemia. Mechanistically, p38 MAPK activation upregulates survival proteins Bcl-2 and B-cell lymphoma-extra-large (Bcl-xL) through cAMP-responsive element-binding protein-dependent transcriptional regulation, while concurrently inducing mitochondrial permeability transition pore opening - a critical determinant of mitochondrial apoptotic signaling. Mitochondrial permeability transition pore opening enables X-linked inhibitors of apoptosis protein family members to bind caspases, directly modulating apoptotic signaling pathways (Figure 3). Experimental data further indicate that frequency-specific EA elevates the Bcl-2/Bcl-2-associated X protein (Bax) and Bcl-xL/Bax ratios, stabilizing mitochondrial membrane integrity post-CIRI through anti-apoptotic protein dominance, thereby conferring neuroprotective efficacy (Cheng et al., 2015). EA exerts dual regulatory effects on astrocytes and microglia through targeted molecular interventions. Specifically, EA suppresses astrocytic S100B secretion — a critical mediator that upregulates inducible nitric oxide synthase (iNOS) expression and drives pathological nitric oxide (NO) overproduction in glial cells at elevated concentrations, ultimately triggering neurotoxic NO diffusion. This coordinated modulation attenuates nitro-oxidative stress propagation and suppresses proinflammatory cytokine release, demonstrating EA’s capacity to disrupt the S100B/iNOS/NO neurotoxic axis while preserving neural homeostasis (Cheng et al., 2014).

While the precise mechanisms underlying EA regulation of redox homeostasis require further elucidation, its capacity to mitigate CIRI through coordinated ROS suppression and enzymatic antioxidant system modulation is well-established. Current evidence reveals substantial pathway convergence among EA’s antioxidant, anti-inflammatory, and anti-apoptotic effects. Critical nodal regulators like the MAPK signaling cascade demonstrate multifunctional therapeutic potential, simultaneously addressing oxidative stress propagation, neuroinflammation amplification, and programed cell death initiation post-CIRI. This mechanistic overlap highlights this regulator as a central role in EA-mediated neural protection, positioning it as a prime translational target for developing multimodal interventions against ischemia-reperfusion pathologies.

MAPK and cytochrome C are relatively important intracellular targets in recent studies, acting by affecting the production of oxidative substances and regulating oxidase production, while in glial cells EA also reduces the production of substances that contribute to oxidative stress, such as S100B.

#### 3.1.3 Neuroinflammation

In CIRI, inflammation is initiated through endogenous signaling, wherein damaged tissues release molecular mediators that activate innate immune responses. Plasma membrane rupture in necrotic cells triggers the extracellular release of substances, including ATP, potassium ions, uric acid, high-mobility group box 1 protein, and S100 calcium-binding proteins. The release of these substances activates multiple inflammasome complexes, including NLRP3 and NALP3, while simultaneously triggering Toll-like receptor-mediated caspase-1 activation. This signaling mechanism propagates inflammatory cascades and induces pyroptotic cell death. ROS, central to oxidative stress, further amplifies inflammation through mechanisms such as oxidized lipoprotein-induced inflammatory signaling (Jayaraj et al., 2019). Key inflammatory mediators including tumor necrosis factor-α (TNF-α), NF-κB, and interleukin-1β (IL-1β) critically regulate inflammatory cascade activation in CIRI (Cao et al., 2021).

EA significantly inhibits inflammatory responses in CIRI. Experimental studies employing focal cerebral ischemia models in male Sprague-Dawley rats demonstrated that EA suppresses NLRP3 inflammasome activation in penumbral tissues post-reperfusion, as quantified by Western blot and immunofluorescence analyses. Mechanistically, EA activates α7 nicotinic acetylcholine receptor signaling to block NLRP3 inflammasome assembly, thereby attenuating neuroinflammation (Jiang et al., 2019). EA directly inhibits the production of major inflammatory factors, including TNF-α, IL-6, IL-1β, and Ccl-2 mRNA, exerting an anti-inflammatory effect within the cell (Ren et al., 2024). It also modulates immune cells such as Tregs and γδ T-cells, which suppress other T-cells, B-cells, and pro-inflammatory factors through this pathway, thereby reducing inflammation (Wang Y. et al., 2023).

Recent studies suggest that EA is closely associated with both anti-apoptotic and anti-inflammatory mechanisms, involving pathways that simultaneously downregulate inflammatory factors and exert inflammatory-trophic effects. For example, in a brain ischemia-reperfusion model, EA treatment was evaluated using cell fluorescent dye and real-time quantitative polymerase chain reaction to assess mRNA levels. The expression of brain-derived neurotrophic factor (BDNF) was measured via western blot or enzyme-linked immunosorbent assay *in vitro*, revealing that EA induced delta-opioid receptor activation and improved cell morphology.

It reduced cell apoptosis and downregulated proinflammatory cytokine expression. Additionally, the delta-opioid receptor inhibited inflammation through the BDNF/tropomyosin-related kinase B pathway (Geng et al., 2020). BDNF has anti-inflammatory effects and can inhibit neuronal apoptosis (Gu W. et al., 2022). However, the specific anti-inflammatory mechanism of EA remains insufficiently studied. Future research clarifying its mechanism of action will enhance the clinical application of this traditional Chinese medicine-based treatment, enabling more targeted therapeutic effects.

#### 3.1.4 Ferroptosis

**FIGURE 4:**
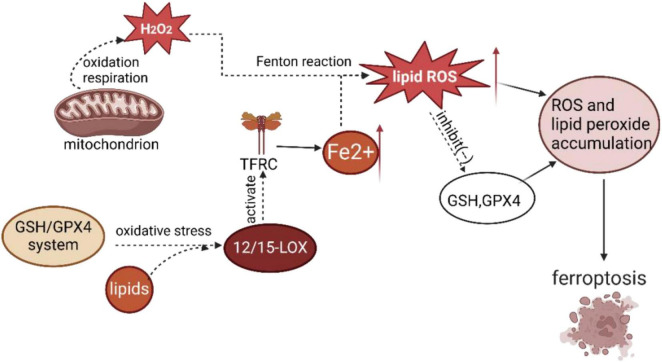
Diagram of the process of cellular ferroptosis.

EA can play a protective role against CIRI and ameliorate it through the inhibition of neuronal ferroptosis. Some studies (Liang et al., 2022; Yao et al., 2021) demonstrate that ferroptosis, an iron-dependent form of programed cell death caused by lipid peroxide accumulation, plays a critical role in the development of neurological diseases. This process is closely related to intracellular GSH deficiency and ROS production. Glutathione peroxidase 4 (GPX4) is a critical inhibitor of ferroptosis, and the GSH/GPX4 system reduces lipid hydroperoxides to 12/15-lipoxygenase (12/15-LOX) under oxidative stress (Liu S. et al., 2023), activating the transferrin receptor (TFRC) to enhance ferrous ion (Fe^2+^) uptake (Lu et al., 2021). GSH biosynthesis is critical for the functional activity of GPX4. When GSH levels are depleted, GPX4 becomes inactivated, leading to ROS accumulation and lipid peroxidation, which at a certain threshold triggers cellular ferroptosis (Ou et al., 2022). Biochemically, the Fenton reaction between excess free Fe^2+^ and hydrogen peroxide, a product of mitochondrial oxidative respiration, triggers a significant increase in ROS, which inhibits GSH synthesis and GPX4 activity. This leads to impaired antioxidant systems, redox imbalances, and a buildup of toxic lipid peroxides, ultimately contributing to the ferroptosis of cells (Jiang et al., 2021; Lei et al., 2019; Li et al., 2020; Xie et al., 2022). The approximate process of ferroptosis described above can be seen in Figure 4.

Li et al. demonstrated that EA has a ferroptosis-inhibitory effect in MCAO rats (Li et al., 2021). The study showed that EA enhances GPX4 and SOD activities, as well as GSH levels while decreasing MDA and iron accumulation. EA was also found to increase the levels of FTH1 and decrease the levels of Tf (transferrin) and TfR1. These results suggest that EA alleviates ICS (ischemic stroke) by inhibiting ferroptosis. Moreover, Wang G. et al. (2023) found that EA significantly reduced the expression of ACSL4 and TFR1, increased GPX4 levels, and inhibited ROS production in MCAO/R model rats. Furthermore, EA treatment improved neurological deficits and reduced infarct volume. These findings suggest that EA may alleviate CIRI by inhibiting ferroptosis. The results from these animal studies indicate that EA holds the potential for mitigating CIRI through ferroptosis inhibition.

GSH, GPX4, and ROS are keys to the occurrence of ferroptosis in neurons. EA can inhibit neuronal ferroptosis after cerebral ischemia/reperfusion and attenuate cerebral ischemia/reperfusion injury by decreasing the content of GSH and ROS and downregulating the expression of GPX4.

#### 3.1.5 Neuronal apoptosis (apoptosis-related protein balance)

EA can exert anti-neuronal apoptosis by regulating the balance of apoptosis-related proteins, thereby demonstrating therapeutic potential in improving CIRI (Ma et al., 2016).

Firstly, EA treatment can prevent neuronal apoptosis by regulating the balance of Bcl-2 and Bax proteins. Bcl-2 and Bax are key proteins in the Bcl-2 family, closely associated with apoptosis. Bcl-2 is a membrane-stabilizing protein that is associated with organelle membranes. By inhibiting free radical production and intracellular calcium overload, this treatment prevents mitochondrial membrane permeability, maintains membrane stability, and blocks cytochrome C release and caspase activation, thereby inhibiting apoptosis. Bcl-2 overexpression reduces cellular damage, while Bax exhibits an antagonistic role, promoting apoptosis. Bcl-2 and Bax can form heterodimers. When Bcl-2 expression is increased, Bcl-2 homodimers are formed, which protect cells. Conversely, when Bax expression is increased, Bax/Bcl-2 heterodimers form, leading to apoptosis (Alam et al., 2022; Moldoveanu and Czabotar, 2020; Wolf et al., 2022). Therefore, the Bcl-2/Bax ratio plays a significant role in determining apoptosis in neurons and other cells. It has been shown that in the MCAO-induced cerebral ischemia/reperfusion rat model, EA treatment significantly enhanced the Bcl-2/Bax ratio, reduced the number of apoptotic cells, and alleviated the degree of neurological impairment. Additionally, EA improved cognitive deficits in ischemia/reperfusion-injured rats by regulating Bcl-2 and Bax expression (Liu et al., 2015). This finding is consistent with the experimental study by Ma et al. (2016), which demonstrated that EA stimulation in MCAO rats up-regulated Bcl-2 protein expression, down-regulated Bax protein expression and significantly reduced the number of apoptotic cells. These results suggest that EA may mitigate neuronal apoptosis following CIRI by modulating the balance between Bax and Bcl-2 proteins.

Secondly, EA can also reduce CIRI by regulating caspase-3 expression, an apoptotic execution factor that exists in normal cells as a zymogen. During cerebral ischemia, caspase-3 activation triggers a series of events, including nuclear factor activation, cytoskeletal protein disruption, and DNA cleavage. These processes lead to cell morphological changes, apoptotic vesicle formation, and ultimately apoptosis (Sahoo et al., 2023; Unnisa et al., 2023; Vatandoust et al., 2023). Meng et al. (2024) demonstrated that EA treatment in MCAO rats significantly upregulated the mRNA and protein expression of the Bcl-2 gene in the ischemic brain, thereby downregulating caspase-3, Bax, and cleaved caspase-3. This led to a reduction in apoptosis of the ischemic brain tissue in stroke rats and attenuation of CIRI in MCAO rats. EA treatment enhances neuroprotection by promoting the expression of protective proteins and inhibiting pro-apoptotic factors, resulting in improved outcomes for cerebral ischemia-reperfusion injury.

**FIGURE 5:**
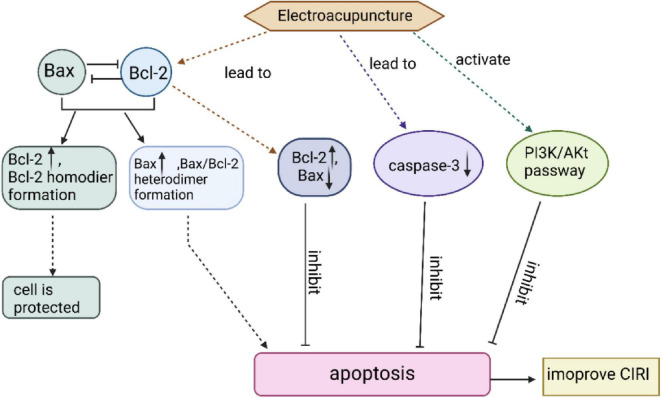
EA can inhibit neuronal apoptosis and ameliorate brain tissue injury after CIRI through three mechanisms.

In addition, studies have demonstrated that EA may inhibit neuronal apoptosis by activating the PI3K/Akt pathway, thereby protecting brain tissue from CIRI (Rao et al., 2025; Wang et al., 2021). Additionally, EA enhances BDNF expression, further exerting anti-apoptotic, neuroprotective effects in CIRI (Geng et al., 2020; Li et al., 2022). These mechanisms are summarized in Figure 5.

Overexpression of Bcl-2 attenuates cell damage, whereas Bax acts in the opposite direction to Bcl-2 and has a pro-apoptotic effect. EA intervention in MCAO rats up-regulated Bcl-2 protein expression and down-regulated Bax protein expression, which significantly reduced the number of apoptotic cells and exerted neuroprotective effects. caspase-3 is an apoptotic execution factor that triggers cell apoptosis. EA can inhibit apoptosis in the ischemic brain tissue of stroke rats by reducing the expression of caspase-3. EA can also activate the PI3K/Akt pathway, causing a series of responses to inhibit neuronal apoptosis after CIRI.

#### 3.1.6 Excitotoxic injury (high calcium permeability of NMDARs)

CIRI leads to excitotoxic injury, primarily due to the excessive release of excitatory amino acids. This process causes neuronal apoptosis and neurotoxic injury, characterized by increased glutamate release. Glutamate (Glu), an excitatory neurotransmitter, activates NMDA receptors (NMDARs). The excessive release of glutamate into the synaptic gap triggers increased calcium influx through NMDARs (NMDA receptors), which is central to the toxic damage (Liao et al., 2023).

**FIGURE 6:**
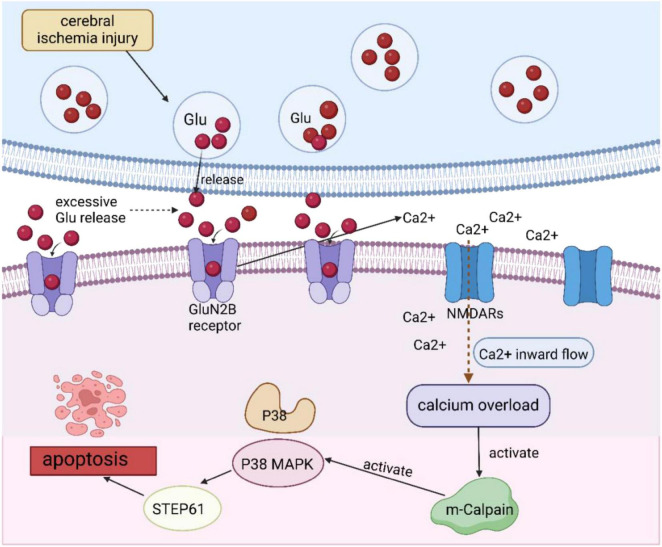
CIRI-induced injury regarding glutamatergic excitotoxicity.

Under pathological conditions such as cerebral ischemia, glutamate is excessively released into the synaptic gap, which then activates the NR2B-containing NMDAR (GluN2B), leading to intracellular calcium overload. The overloaded calcium activates cytoplasmic m-calpain, which triggers the enzymatic activity of STEP61, further inhibits p38 MAPK dephosphorylation, and ultimately leads to apoptosis through signal transduction (Huang W. et al., 2022; Jang et al., 2014). This process is illustrated in Figure 6. Therefore, inhibition of glutamate over-release is very important for mitigating CIRI. It has been reported in the literature (Zhang et al., 2018) that EA administration in MCAO rat models increased NMDAR2A protein expression in the hippocampus compared to the MCAO group. It also reduced Glu and NMDAR2B protein levels and decreased intracellular Ca^2+^ levels in the MCAO rats. This helped protect the hippocampus and attenuate the CIRI caused by MCAO. The mechanism behind this effect includes the reduction of NMDAR2B expression and the decrease of Ca^2+^-related neurotoxicity by inhibiting Glu release. Zhang et al. (2020) demonstrated that EA pretreatment down-regulated the GluN2B expression, reduced m-calpain activity, and inhibited p38 MAPK phosphorylation in hippocampal CA1 neurons. These findings suggest that EA suppresses the GluN2B/m-calpain/p38 MAPK pathway, thereby reducing brain damage following CIRI. The proapoptotic pathway of GluN2B/m-calpain/p38 MAPK effectively reduced the volume of cerebral infarction and the level of neuronal apoptosis after CIRI.

Following CIRI, EA reduced excitotoxicity brought on by calcium overload in brain tissues and prevented glutamate release. Pretreatment with EA may be useful in lowering the amount of cerebral infarction and the degree of neuronal death during CIRI by blocking the GluN2B/m-calpain/p38 MAPK pro-apoptotic pathway.

In addition, NMDARs exhibit two subtypes, NMDAR2A and NMDAR2B, which have opposing roles. NMDAR2A activation promotes neuronal regeneration and protects neuronal function, while NMDAR2B mediates the free radical chain reaction, inducing neurotoxicity and apoptosis (Goldsmith, 2019; Skowrońska et al., 2019). Zhang et al. showed that EA enhanced NMDAR2A expression and down-regulated NMDAR2B expression, thereby alleviating CIRI. This modulation significantly influenced cognitive recovery post-stroke in a rat model (Zhang et al., 2018).

Previous studies have shown that while reducing glutamate release or enhancing glutamate reuptake are effective strategies to counter ischemic injury, most drugs in this category are inefficient in reducing morbidity or mortality and exhibit neurological side effects in stroke clinical trials. Targeted therapies and interventions based on the “NMDAR” subtype hypothesis have also shown limited efficacy and significant side effects in preclinical studies (Mao et al., 2022). Therefore, the current study on EA for CIRI highlights a potential mechanism to address excitotoxic injury, which could address the limitations of existing ischemic stroke therapies. Moreover, EA intervention is free of drug toxicity and has minimal neurological side effects, offering promise for broader clinical application in the foreseeable future.

### 3.2 Promotion effect

#### 3.2.1 Blood-brain barrier

EA intervention may also ameliorate CIRI by protecting the blood-brain barrier. Cerebral ischemia/reperfusion can cause and aggravate cerebral edema by increasing the expression of Aquaporin-4 (AQP4), a water channel protein closely associated with cerebral edema formation (Vandebroek and Yasui, 2020). Taniguchi et al. (2000) found that after focal cerebral ischemia in rats, cerebral edema worsened over time and the expression of AQP4 protein and mRNA was in line with the time of aggravation of cerebral edema. The changes in AQP4 mRNA expression in MCAO were closely related to the changes in edema formation and regression. Xu et al. (2014) demonstrated that EA significantly attenuated the expression of water channel protein AQP4 in the ischemic brain, effectively alleviating cerebral edema symptoms in rats following cerebral ischemia and reperfusion. Studies have also shown that AQP4 is closely related to the BBB (Mueller et al., 2023). Neural astrocyte peduncles contribute to the BBB’s structure by forming a glial border membrane around capillaries, acting as the BBB’s second barrier. This structure aids in regulating water transport, with AQP4 concentrated in the peduncles of glial cells. Under cerebral ischemia, AQP4 expression on astrocyte membranes is enhanced. AQP4 channels open abundantly, leading to cerebral edema formation and progression. Therefore, EA downregulates AQP4 expression, thereby reducing astrocyte peduncle swelling and restricting AQP4 activity. This leads to BBB protection, cerebral edema alleviation, and improved outcomes in CIRI.

Additionally, EA may improve CIRI by down-regulating Matrix metalloproteinase 2 (MMP2)/AQP4/APQ9 expression. MMPs belong to the family of Zn^2+^-dependent enzymes associated with BBB permeability and disruption (Lu and Wen, 2025; Sun et al., 2023), and MMP2 plays an important role in brain water transport homeostasis, as do AQP4 and AQP9. All three are associated with the permeability of the BBB in CIRI. BBB disruption, tissue inflammation, and MMP/AQP up-regulation together trigger cerebral edema/swelling after CIRI. EA mitigates this condition by regulating MMP2/AQP4/AQP9 activity, thereby exerting a neuroprotective effect (Xu et al., 2014).

MMP-9, a collagenase in the MMP family, degrades the extracellular matrix and contributes to BBB disruption during CIRI (Gu Y. et al., 2022). In contrast, some studies have shown that MMP-9 expression in the brain of MCAO rats pretreated with EA was significantly reduced, suggesting that EA pretreatment may maintain the integrity of the blood-brain barrier after injury by inhibiting MMP-9 expression, reduce the brain tissue damage, and improve the cerebral function (Lin et al., 2016).

#### 3.2.2 Angiogenesis

**FIGURE 7:**
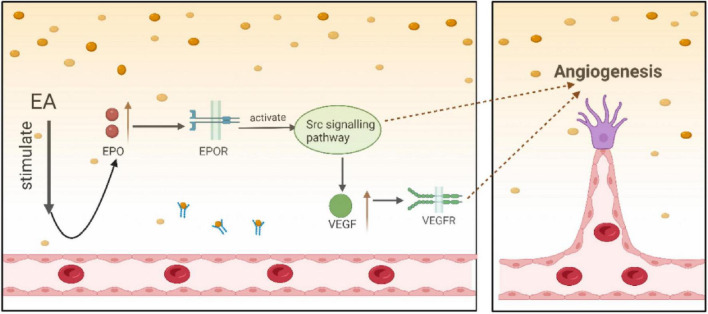
EA activates EPO-mediated pathways to promote angiogenesis.

Following CIRI, the microvasculature of the cerebral cortex is damaged, resulting in neuronal death in the ischemic central zone. Neurons in the ischemic hemiparetic zone are subjected to ischemia and hypoxia, leading to neuronal damage and functional impairments (Wang et al., 2018). In this case, angiogenesis in ischemic areas is crucial for reducing the damage and improving prognosis. Angiogenesis not only increases collateral circulation to reduce ischemia-induced brain damage but also plays a key role in restoring neurological function after ischemic stroke (Shi et al., 2017). Angiogenesis is regulated by several vascular growth factors, including vascular endothelial growth factor (VEGF) and erythropoietin (EPO). Previous studies have shown that VEGF is a key cytokine associated with the differentiation, proliferation, and migration of endothelial cells. By binding to its receptor (VEGFR), VEGF activates downstream signaling pathways and transmits angiogenic signals (Wang H. et al., 2020). Therefore, promoting the expression of VEGF and VEGFR is important in angiogenesis after brain injury. In this regard, studies have shown that EA can promote neovascularization after focal cerebral ischemia/reperfusion by up-regulating VEGF expression, reducing the damage of the vascular wall induced by ischemia/reperfusion, and exerting a protective effect against ischemia/reperfusion-induced brain injury (Zhang W. et al., 2023). EPO exhibits multiple functions, including erythropoiesis promotion, anti-inflammatory, antioxidant, pro-angiogenic, and neurotrophic effects (Alural et al., 2014; Si et al., 2019). EPO activates its function by binding to the EPO receptor (EPOR). Importantly, both EPO and EPOR are expressed in red lineage cells, neurons, astrocytes, and cerebrovascular endothelial cells (Tsiftsoglou, 2021). The non-receptor tyrosine kinase Src plays an important role in angiogenesis, and increased levels of Src phosphorylation have a protective effect against ischemia-induced brain damage (Sun et al., 2021). Wang et al. (2022a) showed that EA increased the expression of EPO, VEGF and P-Src proteins. This effect may involve EPO-mediated activation of the Src signaling pathway and VEGF signaling pathway, thereby promoting angiogenesis. Notably, EPO activates the Src pathway, which further increases VEGF expression (Figure 7), amplifying pro-angiogenic effects. This process facilitates vascular neogenesis, enhancing blood flow and oxygen supply in ischemic brain areas, and alleviates CIRI.

In CIRI rat brain tissue, EA stimulates the VEGF and Src signaling pathways, which are mediated by EPO, to encourage angiogenesis. Moreover, activation of the Src signaling pathway by EPO contributes to the increase in VEGF expression, which further enhances the pro-angiogenic effect and facilitates neovascularization, thereby attenuating CIRI.

Moreover, EA may also promote the mobilization of endothelial progenitor cells (EPCs) to ischemic areas and promote vascular neogenesis. EPCs are a group of naïve endothelial cells with wandering characteristics and capable of further proliferation and directional differentiation. Their main function is to participate in vascular endothelial repair and neointimal formation in ischemic organs (Kong et al., 2018; Shi et al., 2023). After the ischemic injury, EPCs in bone marrow may mobilize, enter the peripheral circulation, and migrate to the ischemic site to participate in vascular endothelialisation and angiogenesis. EA promotes the mobilization of EPCs in the bone marrow and their migration in the peripheral blood, which facilitates angiogenesis after cerebral ischemia/reperfusion and improves injury symptoms (Lu et al., 2013; Xie et al., 2016).

EA’s key advantage in CIRI treatment lies in its ability to stimulate cerebral angiogenesis at injury sites. Unlike existing ischemic stroke treatments, fewer drugs target cerebral angiogenesis. EA offers a promising new therapeutic approach for the clinical treatment of ischemic stroke.

## 4 Conclusion

After thrombolysis or thrombectomy for cerebral ischemic stroke, the infarcted blood vessel is reopened, and the blood supply is restored. However, this process triggers a series of cellular responses that exacerbate the damage to the ischemic tissue, known as CIRI. EA is an enhanced traditional Chinese medicine technique that provides protective mechanisms for the brain. These mechanisms include but are not limited to, modulating neuroinflammatory responses, regulating cellular autophagy, and reducing oxidative stress.

The beneficial effects of EA on CIRI are evident across multiple dimensions, particularly in its antioxidative stress and anti-inflammatory responses. These two effects are interdependent and synergistic, collectively reducing the production of inflammatory factors and mitigating the inflammatory response. Concurrently, EA alleviates the accumulation of ROS and the occurrence of oxidative stress. Although the exact mechanism by which EA regulates autophagy remains unclear and some experimental results appear contradictory, with controlled conditions, a consistent mechanism and pattern can be discerned. Regulation of autophagy in neuronal cells, glial cells, and intracellular organelles has emerged as a significant target for EA therapy. EA can reverse cell death and apoptosis caused by excessive autophagy in neuronal cells after injury while up-regulating autophagy in intracellular mitochondria helps clear cytotoxic substances. Despite the current uncertainty regarding the regulation of autophagy and the selection of autophagic targets, the phenomenon observed in EA therapy is promising and suggests substantial therapeutic potential. Investigating the detailed mechanisms of autophagy regulation by EA is crucial. Autophagy is intricately linked to both antioxidative and anti-inflammatory mechanisms. For instance, autophagy-related pathways can generate essential ROS for the inflammatory response and oxidative stress. These mechanisms are interconnected rather than isolated. By leveraging these amplifying effects, we can identify central targets and substances, such as MAPK and ROS, which interact and regulate related pathways to alleviate CIRI. This integrated approach holds significant promise for enhancing the therapeutic efficacy of EA in treating CIRI.

The mechanisms by which EA improves CIRI are interconnected and multifaceted. Oxidative stress induced by CIRI contributes to cellular ferroptosis, and inhibiting neuronal ferroptosis can mitigate oxidative stress damage. EA promotes angiogenesis, which enhances blood and oxygen supply to brain tissues and helps protect the blood-brain barrier. Therefore, the mechanisms of EA should not be viewed in isolation. However, current studies often focus narrowly on specific pathways or proteins, and most use MCAO rat models, limiting the direct applicability to human treatment. In the design of research experiments, there are issues related to inadequate sample sizes and inconsistencies in EA points and frequencies. In studies examining EA for stroke treatment, animal experiment sample sizes typically range from 30 to 100, which is generally considered small. This limited sample size may not accurately represent the overall effectiveness of EA therapy, leading to restricted applicability of the findings. Additionally, the lack of standardized EA parameters, such as points, frequency, and intensity, complicates comparisons across different studies, thereby impacting the reliability and reproducibility of the results. Future experimental designs should focus on varying acupoints, stimulation durations, and treatment lengths to identify the most effective treatment protocols, thereby controlling EA-related variables and enhancing the study’s quality. Furthermore, future research could increase sample sizes and consider using primate models to improve the experimental animal models. Nonetheless, EA treatment and pretreatment have both demonstrated significant improvement in CIRI in experimental animals, highlighting the potential importance of this research for stroke and related brain diseases. These findings offer new avenues for stroke intervention and treatment, providing hope for improving cognitive and memory functions post-CIRI in humans. Future research should aim to elucidate the precise molecular mechanisms through which EA exerts its protective effects on CIRI. This includes detailed studies on how EA regulates autophagy and its impact on cellular pathways involved in oxidative stress, inflammation, and ferroptosis. Integrating advanced techniques such as RNA sequencing and proteomics can help identify novel targets and pathways influenced by EA.

Additionally, clinical trials are essential to validate EA’s efficacy and safety in human patients. Combining traditional Chinese medicine with modern biomedical research could lead to innovative treatments for stroke and other ischemic brain injuries. Integrating EA into standard clinical practice could revolutionize condition management, significantly improving global patient outcomes. As a traditional Chinese medicine therapeutic method, EA has many unknown mechanisms. Investigating its molecular pathways could uncover new therapeutic mechanisms. EA has advantages, including minimal harm and side effects, a holistic effect on ischemic stroke improvement, and use as a complement to existing therapies for neuroprotection. It is also effective as a post-stroke treatment for neurological injuries and has broad clinical applications. Neuroprotective agents play a crucial role in the current therapeutic approach to ischemic stroke. The current generation of drugs targets inflammatory, excitotoxic, and oxidative stress signaling pathways, including human urinary kallidinogenase and ginkgolides (Paul and Candelario-Jalil, 2021). These mechanisms are analogous to those induced by EA interventions. Consequently, EA can serve as a supplementary enhancement or even an alternative to neuroprotective agents, thereby achieving multi-targeted and multi-effective effects that are challenging to attain with individual neuroprotective agents. It can be used as an enhanced complementary or even alternative treatment to neuroprotective agents, exerting multi-target and multi-effective effects that are difficult to realize by a single neuroprotective agent. This approach reduces the use of chemical drugs and improves the prognosis of stroke patients. There is optimism that EA will be formally introduced into clinical practice, offering new hope to patients worldwide.
